# Association Between the Albumin-Bilirubin (ALBI) Score and All-cause Mortality Risk in Intensive Care Unit Patients with Heart Failure

**DOI:** 10.5334/gh.1379

**Published:** 2024-12-19

**Authors:** Jiuyi Wang, Kai Wang, Guibo Feng, Xin Tian

**Affiliations:** 1 Department of General Medicine, Yongchuan Hospital of Chongqing Medical University, Chongqing 402160, China; 2 Department of Cardiology, The Second Affiliated Hospital of Chongqing Medical University, Chongqing 401336, China; 3 Department of Neurology, The First Affiliated Hospital of Chongqing Medical University, Chongqing Key Laboratory of Neurology, Chongqing 400016, China; 4 Key Laboratory of Major Brain Disease and Aging Research (Ministry of Education), Chongqing Medical University, Chongqing 400016, China

**Keywords:** Albumin-bilirubin, Heart failure, intensive care unit, mortality, Prognosis

## Abstract

**Background:**

The albumin-bilirubin (ALBI) score has demonstrated prognostic value in a range for liver and heart diseases. However, its association with all-cause mortality in intensive care unit (ICU) patients with heart failure remains uncertain.

**Objective:**

This study sought to investigate the relationship between the ALBI score and the risk of all-cause mortality in ICU patients with heart failure.

**Methods and results:**

The ICU patients diagnosed with heart failure were selected from the Medical Information Mart for Intensive Care IV database (MIMIC-IV, version 2.2) and stratified into tertiles according to their ALBI scores. The primary outcome of interest was the occurrence of all-cause mortality within 365 days post-discharge. The analysis encompassed a cohort of 4,239 patients, with Kaplan-Meier curves indicating that individuals with higher ALBI levels exhibited an elevated risk of all-cause mortality (log-rank p < 0.001). Multivariate adjusted Cox regression and subgroup analysis demonstrated that individuals in T2 (hazard ratio (HR) 1.09, 95% CI 0.99–1.21) and T3 (HR 1.17, 95% CI 1.02–1.34) had an increased risk of mortality compared to individuals in T1 (p for trend < 0.001), and each incremental tertile in ALBI was linked to a 10% rise in mortality risk, while each individual unit increase in ALBI was associated with a 36% increase in mortality risk. This relationship was consistently observed across most subgroups, except for using or not using inotropes or vasopressors, different ages, different creatinine levels. The restricted cubic spline (RCS) analysis indicated a linear relationship between ALBI levels and the risk of all-cause mortality.

**Conclusion:**

The ALBI scores are independently associated with the risk of all-cause mortality in ICU patients with heart failure, particularly in those not using inotropes or vasopressors, younger patients, and with lower levels of creatinine. ALBI may help identify high-risk patients and optimize risk stratification in this population.

## Introduction

Heart failure serves as a severe and complex clinical syndrome, its incidence and mortality rates of heart failure have exhibited a rising trajectory on a global scale (1). This condition has emerged as a significant health concern jeopardizing human well-being and quality of life. Notably, a substantial proportion of heart failure cases necessitate intensive care unit (ICU) admission, with a subset of these patients experiencing critical illness and concurrent multiple organ dysfunction syndrome, resulting in an all-cause mortality rate of up to 20%–30% (2). Understanding the evolutionary features of the disease and devising personalized therapeutic approaches are pivotal areas warranting sustained focus and dedication for resolution.

In recent years, there has been a growing focus on the close relationship between liver injury and heart disease, emphasizing the intricate mechanisms of interaction and influence between these organs (3). Studies have shown that 20–30% of patients with acute heart failure have liver dysfunction (4,5). Furthermore, in severe heart failure, the incidence of congestive hepatopathy ranges from 15% to 65% (6). The Child-Pugh scoring system, a commonly utilized tool for predicting the prognosis of patients with liver cirrhosis, incorporates factors including albumin levels, bilirubin levels, prothrombin time/international normalized ratio, ascites volume, and hepatic encephalopathy stage. Despite its widespread use, this system is limited by subjective assessments of ascites and hepatic encephalopathy, lacking standardized and objective evaluation criteria (7). In contrast, the albumin-bilirubin (ALBI) score, which is calculated using serum albumin and bilirubin levels, offers a more objective assessment by treating these biomarkers as continuous variables rather than using critical value thresholds (8). Additionally, in contrast to the Model for End-stage Liver Disease (MELD) score, the ALBI score not only eliminates subjective scoring but also reduces testing costs, making it a more practical tool for prognostic stratification in liver diseases such as liver cancer and cirrhosis. The ALBI score provides clinicians with a valuable basis for making informed decisions in clinical practice (9). Moreover, the prognostic significance of the ALBI score has been increasingly recognized in various non-hepatic conditions, including acute kidney injury and cardiac valve surgery, offering novel insights and approaches for disease prediction and management (10,11). However, the association between the ALBI score and mortality risk in ICU patients with heart failure, as a newly developed scoring system, remains uncertain.

Given the high incidence of liver dysfunction in individuals with heart failure and its potential implications on disease outcomes, this study seeks to elucidate the association between the ALBI score and the risk of 365-day mortality in ICU heart failure patients, providing new research evidence for risk identification in these patients.

## Materials and Methods

### Data source

The study population was obtained from the Medical Information Mart for Intensive Care **IV** database (MIMIC-**IV**, version 2.2), which encompasses data from over 190,000 ICU patients admitted to the Beth Israel Deaconess Medical Center in Boston, Massachusetts, USA, from 2008 to 2019 (12). The database contains a wide range of information, including disease diagnoses, demographic characteristics, vital signs, comorbidities, laboratory tests, medications, surgical procedures, and follow-up survival status. The MIMIC-**IV** database is publicly available and can be accessed after completing the systematic training. The human participant procedures in this study adhered to the ethical guidelines set forth by the institutional and/or national research committee, as well as the 1964 Helsinki Declaration and its subsequent revisions or similar ethical standards. The study conducted a secondary analysis of de-identified patient data from a specified dataset, eliminating the need to obtain informed consent from participants. Due to the publicly accessible and unidentifiable nature of the data, ethical review was considered unnecessary.

### Study population

**Figure 1:**
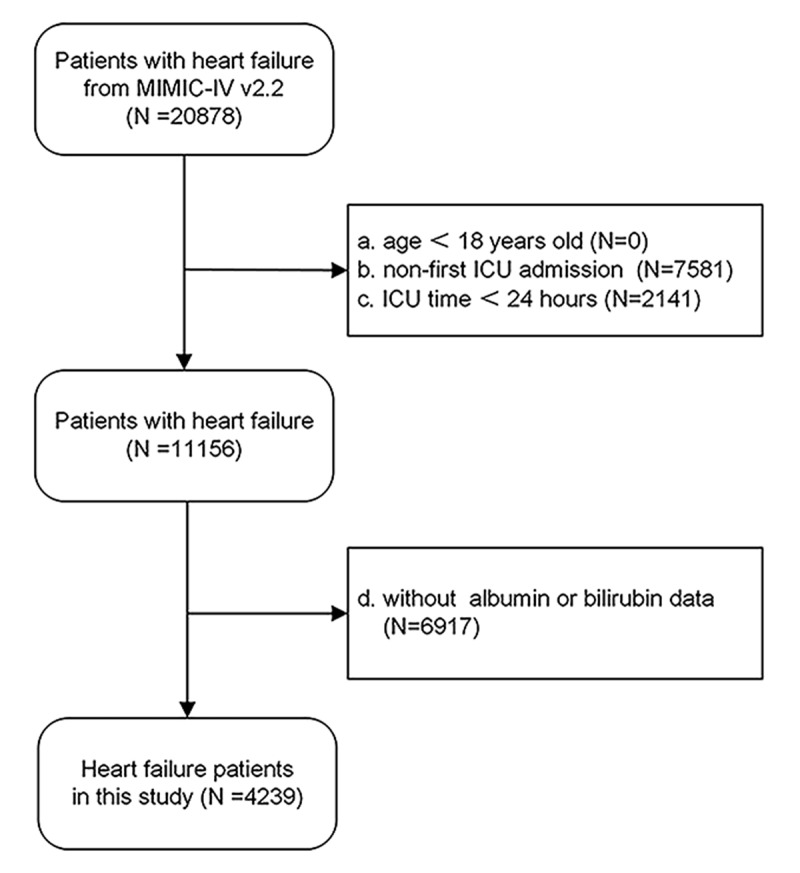
Flow diagram. It presented the inclusion and exclusion of the study participants.

This study applied explicit inclusion criteria. Inclusion criteria: all patients diagnosed with congestive heart failure, identified using International Classification of Diseases, Ninth and Tenth Revision (ICD-9 and ICD-10) codes. Exclusion criteria: a) under 18 years of age, b) not admitted to the ICU on their initial visit, c) with an ICU length of stay less than 24 hours, d) lacking serum albumin or bilirubin data. Ultimately, a total of 4,239 patients were enrolled in the study, as illustrated in the flow chart (Figure 1).

### Data collection and outcome definition

The clinical data were initially gathered within 24 hours after ICU admission, including demographic characteristics (gender, age), vital signs (arterial oxygen saturation (SpO_2_), heart rate, respiratory rate, systolic and mean blood pressure), comorbidities (hypertension, diabetes, chronic obstructive pulmonary disease (COPD)), disease severity scores (APS **III**), laboratory tests (white blood cell count (WBC), platelets, neutrophils, hemoglobin, red cell distribution width (RDW), aspartate aminotransferase (AST), alanine aminotransferase (ALT), alkaline phosphatase (ALP), total bilirubin, total protein, albumin, blood urea nitrogen (BUN), creatinine, anion gap (AG), bicarbonate), medications (dopamine, dobutamine, milrinone, epinephrine, norepinephrine, vasopressin, furosemide), mechanical ventilation, continuous renal replacement therapy (CRRT), and follow-up data (one-year mortality data sourced from the database for all patients). The administration of inotropes or vasopressors was defined as dopamine, dobutamine, milrinone, epinephrine, norepinephrine, phenylephrine, or vasopressin. The ALBI score was computed using these initial measurements and the following formula: ALBI = [albumin (g/L) * –0.085] + [log10 bilirubin (μmol/L) * 0.66] (13). The primary endpoint was all-cause mortality within one year post-discharge.

### Statistical analysis

Normally distributed variables are typically represented using the mean ± standard deviation, while non-normally distributed variables are often depicted using the median (interquartile range) and categorical variables are commonly expressed as percentages. To compare clinical characteristics and mortality rates among the ALBI tertiles (T1, T2, T3), appropriate statistical tests such as the Kruskal-Wallis H test, chi-square test, or analysis of variance were utilized.

Subsequently, Kaplan-Meier survival analysis and the Log-Rank test were employed to evaluate the risk of the primary endpoint in patients with varying ALBI levels.

Next, ALBI was assessed as a categorical (tertiles), ordinal (per-tertile increase), and continuous variable (per-unit increase) in four different Cox regression models to determine the hazard ratios (HRs) and 95% confidence intervals (CIs) for all-cause mortality, with a test for trend. Model 0 included only ALBI without any covariate adjustment. Model 1 was adjusted for age, gender, WBC, hemoglobin, albumin, BUN, and creatinine. Model 2 was adjusted for the variables in Model 1 plus inotropes or vasopressors, CRRT, and mechanical ventilation. Model 3 was adjusted for the variables in Model 2 plus APS **III**. The lowest tertile of ALBI served as the reference category in all models.

Subsequently, restricted cubic spline analysis was employed to capture the dose-response relationship between ALBI and all-cause mortality.

Next, analysis and interaction testing were conducted utilizing Model 3 to evaluate the prognostic relevance of ALBI in various populations. The analysis took into account demographic and clinical variables including gender, comorbidities (hypertension and diabetes), inotropes or vasopressors, CRRT, and mechanical ventilation, along with the visualization of potentially significant interaction terms. Models containing the interaction terms for age × ALBI and creatinine × ALBI were created to assess the interaction between age and ALBI, and between creatinine and ALBI, on a continuous scale, visualized using interaction RCS.

Finally, in order to enhance the reliability of the analysis findings, sensitivity analysis was carried out. Risk ratios (RRs) for the ALBI tertiles were obtained using univariate and multivariate-adjusted Modified Poisson regression, with the outcome being all-cause mortality within 365 days. Statistical analyses were conducted using R software (version 4.3.1; R Foundation for Statistical Computing, Vienna, Austria), with a significance level set at *p* < 0.05.

## Results

### Baseline characteristics

Table 1 displayed the baseline characteristics of the entire study population, consisting of 4,239 subjects categorized by ALBI tertiles, with 1,413 participants in each group. Compared to subjects with lower ALBI levels, those with higher ALBI levels had a lower prevalence of hypertension, higher levels heart rate, WBC, neutrophil, ALT, AST, ALP, total bilirubin, and APS **III** scores; lower levels of SpO_2_, systolic blood pressure, hemoglobin, platelet, and total protein; higher proportion of administration of inotropes or vasopressors, CRRT, and mechanical ventilation. The occurrence of endpoint events was observed in 43.2% (1,831/4,239) of patients with a notable association between higher ALBI scores and increased all-cause mortality rates.

**Table 1:** Baseline characteristics of MIMIC-IV participants according to tertiles of albumin-bilirubin score.


CHARACTERISTICS	OVERALL (n = 4239)	T1 (n = 1413)	T2 (n = 1413)	T3 (n = 1413)	p VALUE

age (years), (mean (SD))	71.79 (14.46)	71.95 (14.61)	72.12 (14.38)	71.31 (14.39)	0.291

male, n (%)	2416 (57.0)	793 (56.1)	794 (56.2)	829 (58.7)	0.297

BMI (kg/m^2^), (mean (SD))	28.88 (6.73)	29.10 (6.79)	29.08 (6.71)	28.47 (6.67)	0.019

diabetes, n (%)	1723 (40.6)	593 (42.0)	592 (41.9)	538 (38.1)	0.055

hypertension, n (%)	3203 (75.6)	1139 (80.6)	1082 (76.6)	982 (69.5)	<0.001

COPD, n (%)	854 (20.1)	298 (21.1)	295 (20.9)	261 (18.5)	0.156

APS III (mean (SD))	57.84 (24.78)	49.63 (20.70)	55.91 (22.44)	67.98 (27.15)	<0.001

heart rate (/min), (mean (SD))	90.76 (21.50)	88.02 (20.18)	90.50 (21.74)	93.77 (22.15)	<0.001

resp rate (/min), (mean (SD))	20.99 (6.35)	20.75 (6.28)	20.96 (6.01)	21.25 (6.74)	0.114

SpO_2_(%) (mean (SD))	96.20 (4.64)	96.47 (4.00)	96.13 (4.74)	96.00 (5.11)	0.022

SBP (mmHg), (mean (SD))	121.59 (25.71)	129.06 (26.57)	121.49 (24.67)	114.21 (23.64)	<0.001

WBC (10^9/L), (mean (SD))	13.05 (10.33)	12.43 (10.32)	13.03 (11.85)	13.70 (8.51)	0.005

hemoglobin(g/dL), (mean (SD))	10.86 (2.51)	11.66 (2.45)	10.88 (2.42)	10.05 (2.39)	<0.001

platelets(10^9/L), (mean (SD))	221.34 (114.61)	231.52 (100.33)	227.26 (116.59)	205.24 (123.95)	<0.001

RDW (%), (mean (SD))	15.78 (2.59)	15.12 (2.29)	15.66 (2.49)	16.55 (2.75)	<0.001

Neutrophils (10^9/L), (mean (SD))	10.46 (6.80)	9.58 (5.98)	10.23 (6.22)	11.58 (7.88)	<0.001

total protein (g/dL), (mean (SD))	5.70 (0.95)	6.28 (0.76)	5.78 (0.79)	5.03 (0.87)	<0.001

Albumin (g/dL), (mean (SD))	3.27 (0.62)	3.87 (0.35)	3.31 (0.28)	2.63 (0.44)	<0.001

ALT (U/L), (mean (SD))	110.45 (439.79)	69.45 (262.97)	117.99 (457.70)	143.90 (546.82)	<0.001

AST (U/L), (mean (SD))	167.62 (733.63)	104.91 (355.38)	167.62 (701.40)	230.32 (994.61)	<0.001

ALP (U/L), (mean (SD))	119.49 (115.74)	100.52 (98.85)	109.81 (77.73)	148.15 (152.05)	<0.001

total bilirubin(mg/dl), (mean (SD))	1.25 (2.44)	0.66 (1.25)	0.95 (1.02)	2.15 (3.74)	<0.001

BUN (mg/dl), (mean (SD))	38.41 (27.84)	36.89 (27.56)	38.50 (27.04)	39.85 (28.83)	0.018

Creatinine (mg/dl), (mean (SD))	1.99 (1.90)	2.01 (2.06)	2.01 (1.95)	1.95 (1.69)	0.625

Drugs, n (%)	1726 (40.7)	404 (28.6)	577 (40.8)	745 (52.7)	<0.001

CRRT, n (%)	333 (7.9)	91 (6.4)	106 (7.5)	136 (9.6)	0.006

Ventilation, n (%)	1716 (40.5)	497 (35.2)	550 (38.9)	669 (47.3)	<0.001

Mortality, n (%)	1831 (43.2)	465 (32.9)	589 (41.7)	777 (55.0)	<0.001


BMI, body mass index; COPD, chronic obstructive pulmonary disease; SpO_2_, saturation of peripheral oxygen; SBP, systolic blood pressure; MCH, mean corpuscular hemoglobin; MCV, mean corpuscular volume; MCHC, mean corpuscular hemoglobin concentration; RDW, red cell distribution width; WBC, white blood cell count; ALT, alanine aminotransferase; AST, aspartate aminotransferase; ALP, alkaline phosphatase; BUN, blood urea nitrogen; Drugs, administration of inotropes or vasopressors; CRRT, continuous renal replacement therapy; drugs, inotropes or vasopressors.

In addition, we presented the information on all patients receiving relevant medications in Supplementary Table 1.

### Kaplan-Meier survival curves

**Figure 2:**
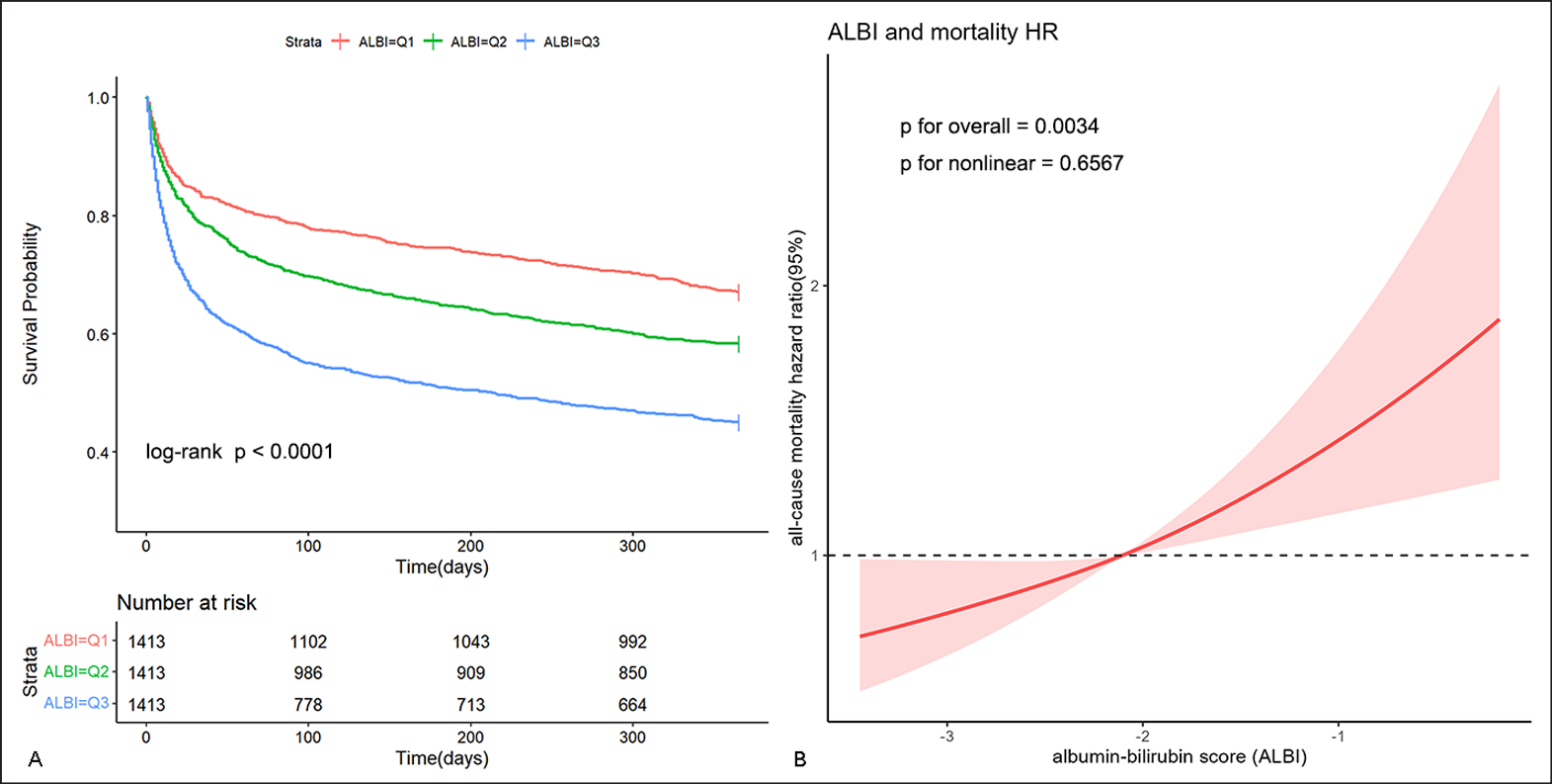
Kaplan-Meier survival curve and restricted cubic spline. Cumulative incidence of all-cause mortality according to ALBI tertiles **(A)** and Cubic spline model of the association between ALBI and risk of all-cause mortality in all patients adjusted with age, gender, WBC, hemoglobin, albumin, BUN, creatinine, inotropes or vasopressors, CRRT, mechanical ventilation and APS III **(B)**.

Kaplan-Meier survival curve analysis was employed to evaluate cumulative survival rates, as depicted in Figure 2A illustrating 365-day cumulative survival rates among three groups categorized by ALBI tertiles. Patients with elevated ALBI levels demonstrated a significantly poorer prognosis relative to those with lower ALBI levels.

### Hazard ratios and dose-response relationship

In Cox regression Model 3, after adjusting for age, gender, WBC, hemoglobin, albumin, BUN, creatinine, inotropes, or vasopressors, CRRT, mechanical ventilation, and APS **III**, the HRs for all-cause mortality in the T2 and T3 groups were: 1.09 (95% CI, 0.95–1.26) and 1.20 (95% CI, 0.99–1.46), respectively, compared to T1 (reference) (p for trend = 0.059) (Table 2). When ALBI was considered as an ordinal variable, after multifactorial adjustment, each tertile increase in ALBI was associated with a 10% increase in mortality risk (HR 1.10, 95% CI 1.00–1.21). When ALBI was included as a continuous variable in the model, after adjusting for confounding factors, each unit increase in ALBI was associated with a 36% increase in mortality risk (HR 1.36, 95% CI 1.14–1.63). The multivariable restricted cubic spline (RCS) regression model demonstrated a linear relationship between ALBI and all-cause mortality risk (Figure 2B) (p for nonlinear = 0.6567), suggesting a rise in all-cause mortality with increasing ALBI levels.

**Table 2:** Cox models for the association between albumin-bilirubin score and all-cause mortality.


ALBI	CASE/TOTAL	MODEL 0	MODEL 1	MODEL 2	MODEL 3
	
TERTILES		HAZARD RATIO			

T1	1413/4239	reference	reference	reference	reference

T2	1413/4239	1.40(1.20, 1.50)	1.13(0.98, 1.30)	1.11(0.97, 1.28)	1.09(0.95, 1.26)

T3	1413/4239	2.10(1.80, 2.30)	1.43(1.18, 1.73)	1.38(1.14, 1.67)	1.20(0.99, 1.46)

p for trend		<0.001	<0.001	<0.001	0.059

Per tertiles increase		1.40(1.40, 1.50)	1.20(1.09, 1.32)	1.18(1.07, 1.29)	1.10(1.00, 1.21)

Per unit increase		1.80(1.60, 1.90)	1.88(1.57, 2.30)	1.83(1.53, 2.20)	1.36(1.14, 1.63)


Model 0: albumin-bilirubin score without adjust; Model 1: age, gender, WBC, hemoglobin, albumin, BUN and creatinine were adjusted; Model 2: the variables in model 1 plus inotropes or vasopressors, CRRT and mechanical ventilation were adjusted; Model 3: the variables in model 2 plus APS III were adjusted.

### Subgroup analysis and interaction effects

**Figure 3:**
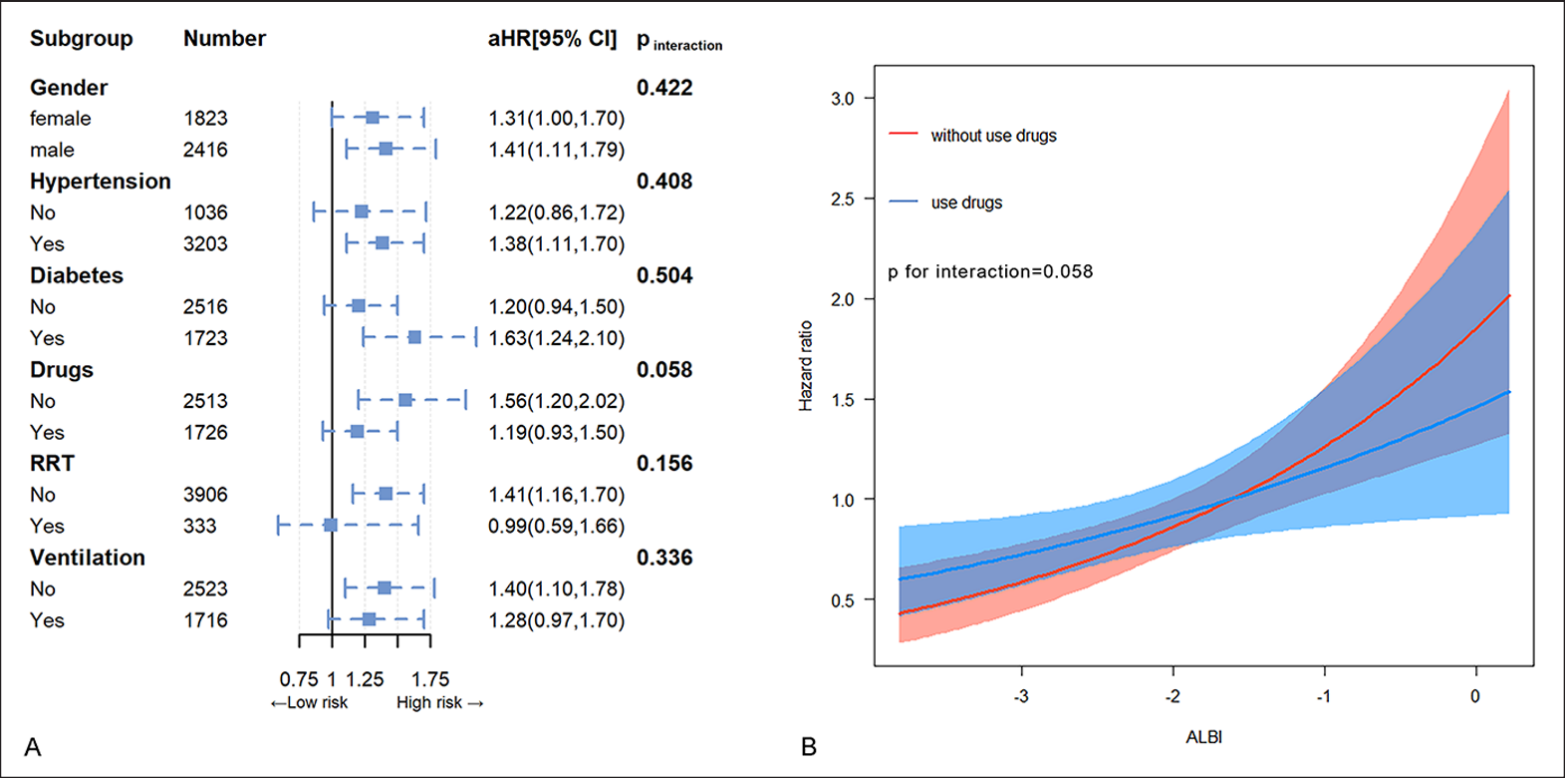
Subgroup analysis. Hazard ratios and error bars delineating 95% confidence intervals from Model 3 by subgroups **(A)** and the trends in the association between ALBI and the hazard ratio for all-cause mortality in subgroups defined by the use of inotropes or vasopressors **(B)**.

Subgroup analysis and interaction effect tests were conducted to evaluate the relationship between ALBI and all-cause mortality within various subgroups defined by gender, diabetes, hypertension, use of inotropes or vasopressors, CRRT, and mechanical ventilation (Figure 3A). The findings indicated a significant interaction between the use of inotropes and vasoactive drugs and ALBI levels in relation to all-cause mortality (p for interaction = 0.058), while no statistically significant interactions were observed in the other subgroups (p for interaction > 0.05). Specifically, among patients not receiving inotropes or vasopressors, each incremental increase in ALBI was associated with a 56% higher risk of mortality (HR 1.56, 95% CI, 1.20–2.02), while in patients receiving inotropes or vasopressors, a mere unit increase in ALBI was found to be correlated with a 19% rise in mortality risk. To illustrate this interaction, graphical representations of the association between ALBI levels and all-cause mortality were generated (Figure 3B). Notably, in patients on inotropes or vasopressors, the escalation of all-cause mortality was gradual as ALBI levels rose, whereas in patients not on inotropes, the initial slow increase in mortality rate with rising ALBI levels shifted to a rapid escalation once ALBI surpassed a specific threshold, resulting in a distinct intersection of the two curves.

**Figure 4:**
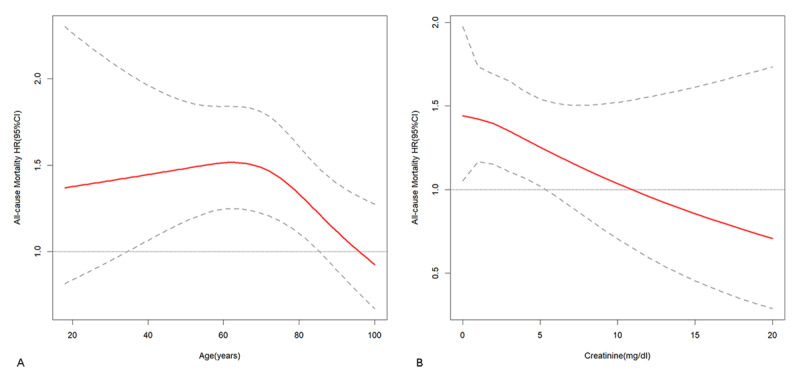
Interaction restricted cubic spline. The trend of the association between ALBI and the hazard ratio (HR) for all-cause mortality with increasing age **(A)** and the trend of the association between ALBI and the hazard ratio (HR) for all-cause mortality with increasing creatinine **(B)**.

Subsequently, an interactive RCS model was employed to investigate the potential interactive association between ALBI and continuous variables (age, creatinine). The findings indicated that the prognostic impact of ALBI scores on patient mortality risk was more prominent at younger ages, diminishing as age increased (Figure 4A). Likewise, a stronger prognostic effect of ALBI scores on mortality risk was observed at lower creatinine levels, with the effect diminishing as creatinine levels increased (Figure 4B).

### Sensitivity analysis

Consistent with the findings of the Cox regression model analysis, in Modified Poisson regression Model 3, compared to T1 (as a reference), the prevalence ratios (PR) for all-cause mortality in the T2 and T3 groups were 1.09 (95% CI, 0.99–1.21) and 1.17 (95% CI, 1.02–1.34), respectively (p for trend = 0.022) (Table 3). Additionally, when ALBI was considered as an ordinal variable, each tertile increase in ALBI was associated with an 8% rise in mortality risk after adjusting for multiple factors. Furthermore, when ALBI was included as a continuous variable in the model and confounding variables were taken into account, each unit increase in ALBI was linked to a 24% increase in mortality risk (PR 1.24, 95% CI, 1.10–1.40).

**Table 3:** Modified Poisson regression models for the association between albumin-bilirubin score and all-cause mortality.


ALBI	CASE/TOTAL	MODEL 0	MODEL 1	MODEL 2	MODEL 3
	
TERTILES		RISK RATIO			

T1	1413/4239	reference	reference	reference	reference

T2	1413/4239	1.27(1.15, 1.40)	1.13(1.02, 1.26)	1.12(1.01, 1.24)	1.09(0.99, 1.21)

T3	1413/4239	1.67(1.53, 1.83)	1.33(1.16, 1.53)	1.30(1.13, 1.49)	1.17(1.02, 1.34)

p for trend		<0.001	<0.001	<0.001	0.022

Per tertiles increase		1.30(1.24, 1.35)	1.15(1.08, 1.24)	1.14(1.06, 1.22)	1.08(1.01, 1.16)

Per unit increase		1.45(1.37, 1.52)	1.53(1.35, 1.73)	1.51(1.33, 1.71)	1.24(1.10, 1.40)


Model 0: albumin-bilirubin score without adjust; Model 1: age, gender, WBC, hemoglobin, albumin, BUN and creatinine were adjusted; Model 2: the variables in model 1 plus inotropes or vasopressors, CRRT and mechanical ventilation were adjusted; Model 3: the variables in model 2 plus APS III were adjusted.

## Discussion

This study investigated the relationship between ALBI scores and all-cause mortality in ICU patients with heart failure. The findings indicated that high levels of ALBI scores were associated with an increased risk of all-cause mortality in ICU patients with heart failure. Notably, ALBI had a stronger prognostic impact on patients who were not utilizing inotropes or vasopressors, were younger in age, and had lower levels of creatinine.

Liver dysfunction, resulting from primary liver diseases and liver congestion and hypoperfusion due to heart failure, frequently result in impaired liver function. The ALBI score, a novel marker for assessing liver function, is believed to represent the liver’s reserve capacity. Studies indicate that the ALBI score may serve as a prognostic tool for liver and heart diseases. A study involving 8,768 patients with primary biliary cholangitis showed that baseline ALBI grade measurement is a straightforward, non-invasive predictor of prognosis in this population (14). Qiao et al. provided confirmation of the ALBI score as a robust independent predictor of mortality in patients with hypertrophic cardiomyopathy (15). Jiang et al. also demonstrated a correlation between the ALBI score and in-hospital mortality in individuals with dilated cardiomyopathy (16). Building upon these findings, this investigation sought to explore the association between the ALBI score and all-cause mortality in ICU patients with heart failure. Through various analytical approaches, such as univariate analysis, multivariate adjustment, and curve fitting, the results consistently indicated a strong relationship between elevated ALBI scores and increased risk of all-cause mortality within one year in ICU patients with heart failure. In addition, sensitivity analysis considering all-cause mortality as a binary outcome, further supported these findings thereby bolstering the credibility of the results. These findings align with those reported in studies conducted by Han and Luo et al. (17,18). Nevertheless, unlike Luo et al.’s investigation, the present study utilized the most recent iteration of the MIMIC database, encompassed a larger patient cohort in the analysis, and undertook a more thorough and comprehensive examination of the linear relationship between ALBI and adverse outcomes. In contrast to Han et al.’s research, the current study specifically focused on ICU patients with heart failure who exhibited greater severity of illness and focused on longer-term all-cause mortality risk. Consequently, this study carries significant practical implications.

Furthermore, this study paid close attention to the prognostic effect of ALBI in different subgroups, which may provide a new perspective on the application of ALBI in the prognostic assessment of heart failure. In the intensive care unit, heart failure patients using inotropes or vasoactive drugs have a higher risk of mortality, elderly or renal dysfunction also have a worse prognosis, which has become a consensus. This also allows this group of patients to receive closer attention from clinicians in the ICU. Moreover, the study meticulously examined the prognostic impact of ALBI in various subgroups, offering a novel insight into the utilization of ALBI for prognostic evaluation in heart failure. It is widely acknowledged that heart failure patients in ICU who require inotropes or vasopressors face an elevated mortality risk, while elderly individuals or those with renal dysfunction experience poorer outcomes (19,20,21,22). Consequently, these patient subgroups naturally warrant heightened vigilance and care from healthcare providers in the ICU setting. Significantly, our subgroup analysis revealed that in patients not receiving inotropes or vasopressors, younger in age, and with lower creatinine levels, the prognostic stratification capability of ALBI was enhanced. This observation may be attributed to the relatively less severe overall health status of these patients, highlighting the prominent role of liver impairment in the advancement of heart failure. These results indicate that ALBI, to some degree, effectively discerns the remaining risk of all-cause mortality in ICU patients with heart failure. This suggests that ALBI may have the potential to garner increased interest from healthcare providers in identifying high-risk patients within this demographic and could serve as a significant element of the risk assessment system. Furthermore, it is noteworthy that the prognostic impact of liver function impairment-related markers (ALBI) on ICU patients with heart failure is influenced by the extent of renal function impairment (serum creatinine levels), indicating a potential interplay among the heart, liver, and kidneys. ALBI appears to play a crucial role in facilitating this essential coordination.

Our study concentrated on all-cause mortality as the primary outcome, revealing that elevated ALBI scores were independently associated with an increased risk of mortality in ICU patients suffering from heart failure. This finding indicates that ALBI, as a marker of liver dysfunction, captures prognostic information that is distinct from conventional cardiac-specific factors. Nonetheless, it is crucial to acknowledge that our study did not directly compare the prognostic efficacy of ALBI with traditional heart failure outcomes or markers, such as cardiovascular mortality, the New York Heart Association (NYHA) classification system, left ventricular ejection fraction (LVEF), N-terminal pro-B-type natriuretic peptide (NT-proBNP), and troponin levels. Future research should undertake a comprehensive evaluation of how ALBI score complements established prognostic factors in predicting mortality and other pertinent outcomes, including cardiovascular mortality, heart failure-related hospitalizations, and quality of life metrics. It is essential to determine whether ALBI offers additional prognostic information beyond these traditional factors and to explore the potential benefits of integrating ALBI with cardiac-specific markers to enhance risk stratification. This would provide a more comprehensive understanding of the clinical utility of ALBI in managing heart failure patients.

From a cost-effectiveness standpoint, the ALBI score presents several potential advantages. Firstly, it is derived from two commonly available and relatively inexpensive laboratory parameters: albumin and bilirubin. This renders the ALBI score an accessible and economically efficient tool for evaluating liver function and prognosis in patients with heart failure, especially when compared to more specialized or invasive diagnostic tests. Secondly, by accurately identifying patients at high risk, the ALBI score may facilitate the optimization of resource allocation and enable the targeting of intensive interventions towards those patients most likely to benefit. However, to comprehensively assess the cost-effectiveness of ALBI score in the management of heart failure, a formal economic analysis is required. This analysis should encompass the costs associated with measuring albumin and bilirubin, potential savings from targeted interventions, and the long-term health outcomes and quality of life of patients stratified by ALBI score. Furthermore, the cost-effectiveness of ALBI score should be compared with other established tools to ascertain its relative value across various clinical settings, ultimately aiming to alleviate the burden of heart failure on healthcare systems globally.

The relationship between ALBI score and prognosis in individuals with heart failure may be attributed to various factors. First, primary liver diseases, as well as liver congestion and hypoperfusion resulting from heart failure, frequently result in hepatic injury. Subsequent impairment of the liver’s metabolic function may exacerbate the disruption of the body’s internal environment, potentially heightening the strain on the heart and worsening heart failure (23). Second, liver dysfunction has the potential to directly impact cardiac function, as evidenced by studies demonstrating that liver damage can trigger cardiomyocyte apoptosis and fibrosis (24). Third, liver dysfunction could indirectly impact the heart by influencing the function of other organs. Research indicates that liver dysfunction frequently coexists with renal dysfunction, from hepatorenal syndrome (25). This syndrome not only disrupts fluid balance and electrolyte metabolism but can also contribute to neuroendocrine disorders, exacerbating cardiac impairment (26). In summary, the ALBI score, which integrates serum albumin and bilirubin levels, provides a comprehensive assessment of liver function. In heart failure patients with liver dysfunction, identifying the underlying cause of liver damage and implementing appropriate interventions may mitigate the adverse effects of liver dysfunction on the heart and optimize secondary prevention strategies.

Our study has several limitations. First, this study is retrospective in nature, leading to the presence of selection and regression bias. The generalizability of the study results to other populations is uncertain. Future research should aim to validate these findings in multi-centered, prospective cohorts. Second, the levels of ALBI were not continuously monitored, potentially impacting the patients’ condition and treatment. Third, despite the utilization of covariates in multifactorial Cox regression models to mitigate confounding factors, residual and unmeasured confounding factors may still influence the study outcomes, such as the duration of heart failure, NT-proBNP. Fourth, detailed information on the stages of heart failure, such as NYHA classification system, was not available. It prevented further analysis on the prognostic performance of the ALBI score across different stages of heart failure. Finally, this study was constrained by data limitations, preventing an in-depth exploration of the specific biological mechanisms underlying the correlation between ALBI and heart, liver, and kidneys. Future research should aim to elucidate the role of ALBI in heart failure at the molecular biology level.

## Conclusion

ALBI scores are independently associated with the risk of all-cause mortality in ICU patients with heart failure with a more pronounced prognostic effect observed in patients not using inotropes or vasoactive medications, younger, and of lower creatinine levels.

## Data Accessibility Statement

The clinical data used to support the findings of this study were supplied by Medical Information Mart for Intensive Care Database IV version 2.2 (MIMIC-IV v.2.2), available at PhysioNet.

## Additional File

The additional file for this article can be found as follows:

10.5334/gh.1379.s1 Supplementary Table 1. Medication information according to tertiles of albumin-bilirubin score.
