# Supplementary material for: Association Between the Albumin-Bilirubin (ALBI) Score and All-cause Mortality Risk in Intensive Care Unit Patients with Heart Failure
Source: Glob Heart. 2024 Dec 19;19(1):97. doi: 10.5334/gh.1379 (PMC11661052; doi:10.5334/gh.1379)
Supplement: Supplementary Table 1. — Medication information according to tertiles of albumin-bilirubin score. [file gh-19-1-1379-s1.pdf]

Supplementary Table 1. medication information according to tertiles of albumin-bilirubin score.

Characteristics	Overall (n=4239)	T1 (n=1413)	T2 (n=1413)	T3 (n=1413)	*p* value
dopamine, n (%)	243 (5.7%)	65 (4.6%)	103 (7.3%)	75 (5.3%)	0.006
norepinephrine, n (%)	1282 (30.2%)	271 (19.2%)	415 (29.4%)	596 (42.2%)	<0.001
dobutamine, n (%)	181 (4.3%)	34 (2.4%)	74 (5.2%)	73 (5.2%)	<0.001
phenylephrine, n (%)	508 (12.0%)	129 (9.1%)	144 (10.2%)	235 (16.6%)	<0.001
epinephrine, n (%)	202 (4.8%)	50 (3.5%)	63 (4.5%)	89 (6.3%)	0.002
vasopressin, n (%)	319 (7.5%)	47 (3.3%)	73 (5.2%)	199 (14.1%)	<0.001
milrinone, n (%)	89 (2.1%)	26 (1.8%)	33 (2.3%)	30 (2.1%)	0.654
β-blocker, n (%)	1809 (42.7%)	730 (51.7%)	598 (42.3%)	481 (34.0%)	<0.001
ACEI/ARB, n (%)	128 (3.0%)	70 (5.0%)	36 (2.5%)	22 (1.6%)	<0.001
furosemide, n (%)	1840 (43.4%)	664 (47.0%)	663 (46.9%)	513 (36.3%)	<0.001

ACEI, angiotensin-converting enzyme inhibitor; ARB, angiotensin II receptor blockers.
